# Supplementary material for: Cloud BioLinux: pre-configured and on-demand bioinformatics computing for the genomics community
Source: BMC Bioinformatics. 2012 Mar 19;13:42. doi: 10.1186/1471-2105-13-42 (PMC3372431; doi:10.1186/1471-2105-13-42)
Supplement: Additional file 1 — Supplementary 1 Cloud BioLinux software documentation in the form of a mini, self-contained website. Users need to download and uncompress the .zip file, and open through a web browser the "index.html" file available on the main directory. (ZIP 1823 kb). [file 1471-2105-13-42-S1.ZIP › Cloud-BioLinux-Package-Documentation/docs/phred.html]

Bio-Linux Software Documentation Pages

Back to search form

## phred

|  |  |
| --- | --- |
| Name | phred |
| Description | **Phred** reads DNA sequencing trace files, calls bases, and assigns a quality value to each called base.  **Phred** is required and/or recommended by some packages available on Bio-Linux such as pregap4 (Staden), stars, and trace2dbest. Phred is an integral part of the phred/phrap/consed trio of packages.  The bio-linux-phred package now comes with both the standard, tried and tested version of phred (phred\_0.020425.c), as well as a beta version (phred\_0.071220.c).  To run standard phred, just type `phred` To run the beta version of phred, just type: `phred_0.07`  Please note that there are a number of differences between the two versions of phred, one of which is that the new version can handle data from additional sequencing machine types. The original version recognises these machine types:  ABI\_373\_377, MolDyn\_MegaBACE, ABI\_3700, LI-COR\_4000, Beckman\_CEQ\_2000, ABI\_3100  In addition to having some new flags and functionality, the new phred version recognises:  ABI\_373\_377, ABI\_3700, ABI\_3730, ABI\_3100, ABI\_3130, Beckman\_CEQ\_2000, LI-COR\_4000, MolDyn\_MegaBACE  **Phred**, **phrap** and **consed** are ***licensed software*** and thus are ***not*** installed by default on Bio-Linux. These programs are free to academic researchers but you must obtain a license. Further information on this is available on the official website for phred, phrap and consed. |
| Homepage | http://www.phrap.org |
| Remote Documentation | http://www.phrap.org/phredphrapconsed.html   http://www.phrap.org/phredphrap/phred.html |
